# Cocaine: An Updated Overview on Chemistry, Detection, Biokinetics, and Pharmacotoxicological Aspects including Abuse Pattern

**DOI:** 10.3390/toxins14040278

**Published:** 2022-04-13

**Authors:** Rita Roque Bravo, Ana Carolina Faria, Andreia Machado Brito-da-Costa, Helena Carmo, Přemysl Mladěnka, Diana Dias da Silva, Fernando Remião, on behalf of The OEMONOM Researchers

**Affiliations:** 1 UCIBIO—Applied Molecular Biosciences Unit, Laboratory of Toxicology, Department of Biological Sciences, Faculty of Pharmacy, University of Porto, 4050-313 Porto, Portugal; up201109020@up.pt (R.R.B.); carolinamfaria@sapo.pt (A.C.F.); andreia.machado2403@gmail.com (A.M.B.-d.-C.); helenacarmo@ff.up.pt (H.C.); 2 Associate Laboratory i4HB—Institute for Health and Bioeconomy, Faculty of Pharmacy, University of Porto, 4050-313 Porto, Portugal; 3 TOXRUN—Toxicology Research Unit, University Institute of Health Sciences, IUCS-CESPU, Rua Central de Gandra, 1317, 4585-116 Gandra PRD, Portugal; 4 Department of Pharmacology and Toxicology, Faculty of Pharmacy, Charles University, 500 05 Hradec Králové, Czech Republic; mladenkap@faf.cuni.cz

**Keywords:** sympathomimetics, crack, pharmacokinetics, pharmacodynamics, toxicity, cocaine hydrochloride, drug abuse, drug analysis

## Abstract

Cocaine is one of the most consumed stimulants throughout the world, as official sources report. It is a naturally occurring sympathomimetic tropane alkaloid derived from the leaves of *Erythroxylon coca*, which has been used by South American locals for millennia. Cocaine can usually be found in two forms, cocaine hydrochloride, a white powder, or ‘crack’ cocaine, the free base. While the first is commonly administered by insufflation (‘snorting’) or intravenously, the second is adapted for inhalation (smoking). Cocaine can exert local anaesthetic action by inhibiting voltage-gated sodium channels, thus halting electrical impulse propagation; cocaine also impacts neurotransmission by hindering monoamine reuptake, particularly dopamine, from the synaptic cleft. The excess of available dopamine for postsynaptic activation mediates the pleasurable effects reported by users and contributes to the addictive potential and toxic effects of the drug. Cocaine is metabolised (mostly hepatically) into two main metabolites, ecgonine methyl ester and benzoylecgonine. Other metabolites include, for example, norcocaine and cocaethylene, both displaying pharmacological action, and the last one constituting a biomarker for co-consumption of cocaine with alcohol. This review provides a brief overview of cocaine’s prevalence and patterns of use, its physical-chemical properties and methods for analysis, pharmacokinetics, pharmacodynamics, and multi-level toxicity.

## 1. Introduction

Cocaine is a naturally occurring sympathomimetic alkaloid from the plant *Erythroxylon coca* that has been used as a stimulant, by chewing the leaves or brewing teas, in South America for over 5000 years. Cocaine was firstly isolated from the leaves in the mid-1800s and was at that time considered safe and used in toothache drops, nausea pills, energy tonics, and the original ‘Coca-Cola’ beverage [1,2]. Currently, it is found in one of two forms for (ab)use: Cocaine hydrochloride (also known as ‘coke’, ‘blow’ or ‘snow’), a fine white crystalline powder, which is soluble in water and consumed mainly through the intranasal route (‘sniffing’/‘snorting’), orally or intravenously; or as a free base (resulting from reaction of cocaine hydrochloride with ammonium or baking soda), commonly known as ‘crack cocaine’ or simply ’crack’, and typically consumed via inhalation (the solid mass is cracked into ‘rocks’ that are smoked, using glass or makeshift pipes) [2,3].

Cocaine abuse remains a significant public health problem with serious socio-economic consequences worldwide [4]. According to the most recent World Drug Report, 0.4% of the global population aged 15–64 reported cocaine use in 2019—this corresponds to approximately 20 million people [5]. The latest edition of the European Monitoring Centre for Drug and Drug Addiction (EMCDDA) Drug Report states that it remains the second most abused substance in the European Union, second only to cannabis [6]. Furthermore, despite the global COVID-19 pandemic, European authorities have intercepted at seaports growing amounts of cocaine in 2020 [5]. All the while, case reports detailing the harmful consequences of cocaine use abound [7,8,9,10,11,12,13,14,15,16,17,18,19,20].

The present review aims to provide an informative overview of the available data on cocaine physicochemical properties and detection methods, pharmacodynamics, pharmacokinetics, effects and toxicity, patterns of abuse as well as its prevalence.

## 2. Results and Discussion

### 2.1. Natural Occurrence and Chemical Characterisation of Erythroxylum coca

**Figure 1:**
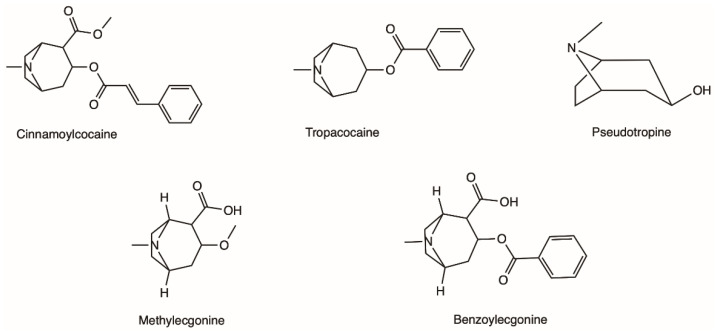
Examples of different alkaloids that can be found in the leaves of the coca plant.

The coca shrub, from which cocaine is extracted, is a plant of the genus *Erythroxylum* that grows in Central and South America, and it has over 250 identified species, of which the two most important are *E. coca* and *Erythroxylum novogranatense*; however it is from *E. coca* Lam. var. *coca* (also referred to as ‘Bolivian’ or ‘Huanuco’ coca) that the majority of cocaine supply is extracted [21,22]. The coca plant presents large, thick, dark green leaves with an elliptical shape and a somewhat sharp apex, and has small red fruits [23]. Circa 18 different alkaloids can be found in the leaves of the coca plant, such as cinnamoylcocaine, tropacocaine, methylecgonine, benzoylecgonine (BE) and pseudotropine—all of these are significantly less euphoric and less toxic than cocaine (Figure 1) [21,22].

Coca leaves have been traditionally used by the indigenous Andean populations and were/are consumed mostly by chewing; coca leaves as a part of religious occasions and other celebrations by the Inca, as well as employed for medicinal purposes [22]. It was from the coca leaves that Albert Niemann first isolated cocaine in 1859–1860 [21,24]. A study from one hundred years later found that dry leaves of *E. coca* var. *coca* have around 6.3 mg of cocaine per gram of plant material [25].

### 2.2. Physicochemical Properties of Cocaine and Analytical Methods for Identification

Cocaine is a tropane alkaloid with weak basic properties. In the free base form, cocaine is unionised and insoluble in aqueous medium, displaying a boiling point of 187 °C; while its ionised hydrochloride salt is readily dissolved in water and presents high stability at very high temperatures, as such, it does not volatilise in the smoke. Table 1 summarises a few of the physical and chemical properties of cocaine [26,27].

One of the most employed methods of detection for cocaine are immunoassays, a fast method that allows a qualitative presumptive assessment of the drug in the biological matrix tested (e.g., blood, urine). However, as this type of test is subjected to a certain degree of false positive and false negative results, a confirmatory quantitative method should be employed afterwards [28,29,30]. Cocaine can be detected in both biological and non-biological matrices through chromatographic methods. A number of reports in the literature describe the detection of cocaine, and its metabolites, in human urine samples [31,32,33,34,35] but also in blood/plasma or hair [36,37,38]. A comparison of methodologies between gas chromatography coupled with mass spectrometry (GC-MS) and high-performance liquid chromatography (HPLC) for the analysis of human urine reported similar outcomes [39]. This allowed laboratories to resort to HPLC for sample examination without such a demanding pre-treatment. Kintz et al. first described a GC-MS method suitable for the simultaneous identification of the ‘crack’-specific metabolite, anhydroecgonine methyl ester (AEME), as well as the parent drug cocaine and additional metabolites (BE, ecgonine methyl ester (EME) and cocaethylene (CE)) in plasma, saliva, urine, sweat and hair samples [37]. This method was successfully applied to 1 mL samples of urine, saliva and plasma, 50 mg of hair, and sweat extracted from a sweat patch, yielding limits of detection for AEME of 1 ng/mL for the first three matrices (with a linearity range of 5–1500 ng/mL), 0.1 ng/mg for the hair (with a linearity range of 0.2–25 ng/mg) and 0.5 ng/patch (with a linearity range of 2–100 ng/patch). Recently, published research by Fernandez et al. described a validated method for the detection of cocaine and several of its metabolites in 0.5 mL of urine samples, resorting to a simple derivatization step following a solid phase extraction (SPE)—the method achieved low limits of quantification from 2.5 to 10 ng/mL by using GC-MS with electron ionization [40]. Liquid chromatography coupled with tandem mass spectrometry (LC-MS/MS) was also successfully employed to detect cocaine and BE both in urine and oral fluid [41], as it was ultra-performance liquid chromatography coupled with tandem mass spectrometry (UPLC-MS/MS) in dried blood samples [42]. Of note, cocaine in blood/plasma samples may undergo spontaneous and enzymatic hydrolysis to its metabolite BE, if the samples are not treated with a pseudocholinesterase (PChE) inhibitor, such as sodium fluoride [24]. As such, samples suspected to contain cocaine should be adjusted to pH 5 with acetic acid and refrigerated at 4 °C or frozen to increase stability of the drug, although some degradation occurs along the time, even at −20 °C.

### 2.3. Legal Status

Since 1961, the International Single Convention on Narcotic Drugs has internationally ruled the recreational use of cocaine as a crime [43]. In the United States, in 1970, the Comprehensive Drug Abuse Prevention and Control Act established cocaine as a Schedule II drug (a substance that has high abuse potential but has medical use in specific instances), a definition the Drug Enforcement Administration (DEA) has been kept to this day [1,44]. The state of Oregon is the only state where, currently, the possession of cocaine (and other drugs) for personal use is decriminalised for amounts under 2 g [45]. In the United Kingdom, the Misuse of Drugs Act, enacted in 1971, made it illegal to possess a Class A drug, such as cocaine [46]. In Europe, cocaine is also generally illegal to possess, sell and transport, as is cultivating coca plant. However, the use of cocaine has a few legal exceptions, such as in Portugal, which has decriminalised the use of major illegal drugs such as cannabis, cocaine and heroin, within the respective threshold amount (also 2 g for cocaine) [47]. The Netherlands has also implemented a decriminalisation model in 1976, where no legal or administrative sanctions apply in the case of possession of any drug (within the threshold amounts of 0.5 g) [48]. Concerning the South American countries, Bolivia has a decriminalisation regime for possession and cultivation of coca leaf; while Chile, Colombia, Peru, Uruguay, Paraguay and Argentina have decriminalised the possession of cocaine when it is proved to be for personal use and meets a set threshold amount [45].

### 2.4. Prevalence, Patterns of (Ab) Use and Public Health Concerns

The most recent data regarding prevalence of cocaine use shows that, in the European Union, 1.2% of adults between the ages of 15 and 64 years have used cocaine in the past year [6]. In South America, the percentage of users in 2019 was almost identical (1%) to that observed in Europe for the same age range, while in North America the prevalence increased to 2.1% [5]. Cocaine is produced mainly in Bolivia, Colombia, and Peru, and from there it is trafficked to intermediate or final destinations. The 2021 edition of the United Nations Office on Drugs and Crime (UNODC) World Drug Report details that 1436 tonnes of cocaine were seized globally in 2019, representing a 9.6% increase when compared to the previous year, and 83% of the seizures took place in the Americas (North, Central and South) [5]. Likewise, the European Drug Report of 2021 informs of a record 213 tonnes seized in the European Union in 2019, accompanied by an increase in product purity and in the number of individuals seeking specialised treatment for cocaine abuse [6].

In Europe, the majority of cocaine users seeking treatment are males of a mean age of 35, whose age of first use averages at 23; have a use frequency of 2–6 days per week; and choose sniffing as their form of intake [6]. Differences in the level of social integration of powder cocaine and ‘crack’ cocaine users are also detailed in the UNODC’s World Drug Report of 2021, albeit these disparities have been fading out in recent years. Users with stable living and work conditions have more sporadic use patterns, consuming cocaine in recreational/nightlife contexts, tending to prefer powdered cocaine, and selecting the intranasal route more often than other ways. On the other hand, users with unstable living and employment conditions tend to select ‘crack’, consume it by inhalation, and have a more frequent use pattern than the first user group [49]. In fact, ‘crack’ use is more likely to result in chronic and heavy use, as it is often associated with specific social groups (e.g., homeless individuals, sex workers) and systemic violence. Polydrug abuse is a frequent practice by both subsets of cocaine users; however, it is more common in the latter group rather than the former, particularly in conjunction with alcohol and heroin (see section ‘Polydrug Use’) [5,50], but also with nicotine and cannabinoids.

In the United States, according to data gathered since the year 2011, reported by the Substance Abuse and Mental Health Services Administration, cocaine was the most common drug of abuse that resulted in hospital treatment, with 505,224 Emergency Department visits (40.3% of all reported drug-related visits), which translates to a rate of 162 visits per 100,000 individuals [51]. A specialised 2021 report by the EMCDDA concerning drug-related deaths in Europe stated that there has been a rise in intravenous cocaine hydrochloride use, as well as the (intravenous) use of ‘crack’, which translates to an increase in cocaine-related emergency department visits and deaths, with the number of fatalities increasing year after year [52].

### 2.5. Pharmacokinetics

#### 2.5.1. Absorption

As previously mentioned, the form of cocaine the user choses (cocaine salt or the free base), the route of administration and patterns of use vary. By virtue of its hydrophilicity, cocaine hydrochloride is generally consumed by ‘snorting’ [53,54]. ‘Crack’ cocaine is generally the only form of cocaine that is smoked—this is due to the fact that cocaine hydrochloride has an elevated boiling point and does not vaporise at the temperatures of combustion [24]. These routes that involve the respiratory system tend to be favoured for both forms of cocaine, as they allow for the stimulant to reach the brain circulation in around 6 to 8 s; the inhalation route presents higher peak plasma concentrations that are reached faster when compared to intranasal administration [53,54]. It should be noted that, for the intranasal route, the vasoconstrictive properties of cocaine slow down the drug’s own absorption, causing a 60-min delay of peak plasmatic concentrations [54]. In terms of bioavailability, the inhalation route has the greatest bioavailability, which surpasses 90%, while the intranasal route has roughly 80% [24]. Regarding time to peak effects and duration of action, inhalation yields peak stimulation within 1 to 3 min after dosing, the stimulus lasting between 5 and 15 min [24]. The intranasal route determines a longer effect, ranging between 15 and 30 min [53].

Intravenous administration of cocaine hydrochloride, by dissolving the powder in an aqueous medium, is also used by consumers [53,54]. Compared with the inhalation and intranasal routes, when cocaine is administered intravenously it takes twice as much time to reach the brain circulation and the peak plasma concentrations are higher and reached faster [53,54]. The bioavailability is closer to the inhalation route [24]. In 1995, Edward Cone published a study directly comparing the pharmacokinetics of cocaine by inhalation, intranasal and intravenous administration (at 42 mg ‘crack’ cocaine, 32 mg cocaine HCl, and 25 mg cocaine HCl, respectively) using the same test subjects for the different routes (cross-over design), and concluded that the peak plasma concentrations were reached within 5 min through intravenous injection and inhalation, whereas the intranasal route took approximately 50 min [55].

Cocaine hydrochloride can also be administered orally, or applied to mucous membranes of the mouth, vagina or rectum [53,54]. Oral administration is associated with the lowest bioavailability, presenting a slower and more erratic absorption. The low degree of bioavailability in this case is explained by gastric breakdown and intestinal metabolism [4,24]. This administration route is often associated with a delayed onset of effects, having the longest effect duration (between 1 and 2 h) [53].

Coca chewing, as an alternative method to consume cocaine, greatly favours sublingual absorption. However, this amounts to smaller doses of the drug when compared to the use of cocaine powder; while the maximum total 24-hour dose of cocaine attained by chewing coca leaves is situated around 200–300 mg (and given that absorption of cocaine will not be 100%, the actual value will certainly be much lower), the average ‘line’ of cocaine hydrochloride has between 20 and 50 mg of the drug, and users will frequently ‘snort’ multiple ‘lines’ in one session. As such, the dose of cocaine administered by users in a recreational session can be significantly greater and the drug whole blood concentration nearly 50 times greater [21]. Holmstedt et al. reported that chewing powdered coca leaves (7–20 g) containing 17–48 mg of cocaine, originated peak plasma concentrations of 11–139 ng/mL cocaine within 0.4–2 h [56]. In addition, in two reported cases of sublingual cocaine HCl use (following neutralization with bicarbonate to increase its absorption and to reduce acidity), a slower onset of action, a later peak effect and a longer duration of action were described when compared with an intravenous route [57]. One other way of consuming coca leaves is through the brewing of teas. Jenkins et al. reported that tea bags containing an average of 4.86 mg (leaves from Bolivia) and 5.11 mg (leaves from Peru) of cocaine per bag, when brewed, produced teas with 4.29 mg and 4.14 mg of cocaine, respectively [58].

#### 2.5.2. Distribution

Cocaine has a fast disposal to the tissues, with a distribution volume ranging between 1 and 3 L/Kg [53,59]. Cocaine binds to albumin and α1-acid glycoprotein at a rate of around 90% and can be found at the highest concentrations in the brain, spleen, kidney, and lungs, followed by blood, the heart and muscle tissue [60]. The average half-life of cocaine is between 40 and 90 min, which may vary depending on the route of administration (shorter for intravenous route, longer for insufflation).

#### 2.5.3. Metabolism

**Figure 2:**
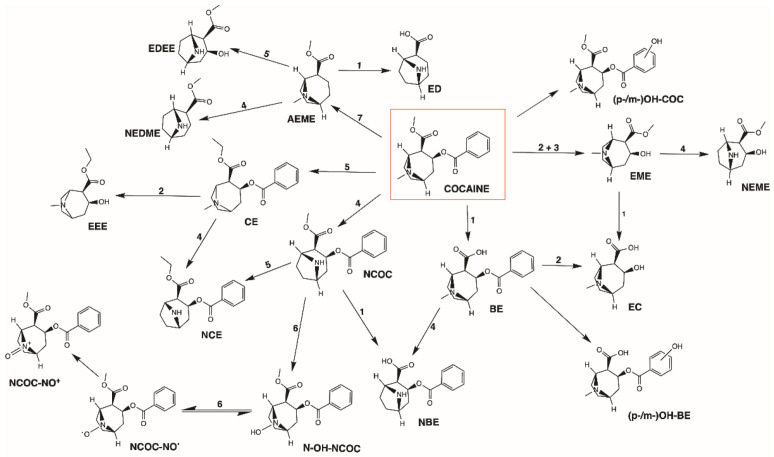
Metabolic pathways of cocaine. Cocaine is mainly metabolized through hydrolysis into benzoylecgonine (**BE**) and ecgonine methyl ester (**EME**), both of which can be further hydrolysed to ecgonine (**EC**). Cocaine may also undergo hydroxylation to yield para-/meta-hydroxycocaine (**p-/m-OH-COC**). Another minor metabolic reaction is the *N*-demethylation of cocaine to norcocaine (**NCOC**). In the presence of ethanol (**EtOH**), cocaine will undergo transesterification and form cocaethylene (**CE**). **AEME**, anhydroecgonine methyl ester; **CYP450**, cytochrome P450; **ED**, ecgonidine; **EDEE**, ecgonidine ethyl ester; **EEE**, ecgonine ethyl ester; **FADM,** flavin adenine dinucleotide-containing monooxygenase; **hCE_1_**, human carboxylesterase type 1; **hCE_2_**, human carboxylesterase type 2; **NBE**, norbenzoylecgonine; **NCE**, norcocaethylene; **NCOC-NO^•^**, norcocaine nitroxide; **NCOC-NO^+^**, norcocaine nitrosonium; **NEDME**, norecgonidine methyl ester; **NEME**, norecgonine methyl ester; **N-OH-NCOC**, N-hydroxy-norcocaine; **(p-/m-)OH-BE**, (para-/meta-)hydroxybenzoylecgonine; **PChE**, pseudocholinesterase.

Cocaine yields two main metabolites: EME and BE, which may both undergo further hydrolysis into ecgonine (EC). EME is a pharmacologically inactive metabolite formed in plasma and in the liver through the action of PChE and carboxylesterase type 2 (hCE_2_), respectively. The other major inactive metabolite of cocaine for all routes of administration, BE, can be formed spontaneously at physiological pH [53] or in the liver by hCE_1_ [61,62]. Cocaine may also undergo *N*-demethylation by cytochrome P450 (CYP) enzyme CYP3A4, generating norcocaine (NCOC), a highly hepatotoxic metabolite capable of crossing the blood–brain barrier (BBB). NCOC accounts for approximately 5% of the absorbed cocaine and was described as a more potent local anaesthetic and more effective at inhibiting noradrenaline uptake by brain synaptosomes than the parent drug [24,63]. BE and EME may undergo further *N*-demethylation by CYP, producing norbenzoylecgonine (NBE) and norecgonine methyl ester (NEME), respectively [62]. Additional minor metabolites are meta-hydroxybenzoylecgonine (*m*-OH-BE), para-hydroxybenzoylecgonine (*p*-OH-BE), meta-hydroxycocaine (*m*-OH-COC), ecgonidine (ED) and norecgonidine methyl ester (NEDME) [32,64]. Figure 2 summarises the metabolic pathways of cocaine.

Of particular relevance, the co-consumption of cocaine and alcohol leads to the formation of CE, a transesterification product of both drugs. Interestingly, it is only produced in vivo via catalysis by hCE_1_ [65]. Harris et al. carried out a study where 10 human subjects received intravenous administrations of cocaine (at 0.3, 0.6 and 1.2 mg/Kg) and a single oral dose of ethanol (at 1 g/Kg), 1 h prior to cocaine intake [66]. They demonstrated that 17% of the intravenous cocaine dose was converted into CE and that ethanol ingestion decreased urinary levels of BE. Another study, resorting to 10 experienced cocaine users, demonstrated that the percentage of CE originated through oral administration of cocaine was larger than that produced by intravenous administration and inhalation (34 ± 20% vs. 24 ± 11% and 18 ± 11%, respectively) [67]. CE is an active metabolite that displays pharmacological activity, with a longer average half-life (148 ± 15 min) than cocaine [24]. Some studies report that it may be more lethal and induce graver acute toxic reactions, also producing a greater increase in heart rate, compared to cocaine [67,68]. CE is used as a biomarker for concomitant use of alcohol and cocaine, which can be detected either in urine to determine recent use, or in hair for chronic exposure [69,70].

Smoking ‘crack’ leads to the formation of another biomarker of exposure, AEME, which is the main product of cocaine’s thermal degradation [71]. In vitro and in vivo studies show that AEME appears to have effects on the cardiovascular system, by acting as a muscarinic agonist [72]. Furthermore, neurotoxic effects were also reported for this metabolite [71,73]. AEME can be further hydrolysed by hCE_1_ into ED, or into ecgonidine ethyl ester (EDEE) when alcohol is present [62]. The determination of AEME and ED in different biological fluids has been proposed as a biomarker for ‘crack’ use [32,60]. The application of the analytical method of Kintz et al. [37] to genuine ‘crack’ users’ samples, allowed identification of AEME in urine (90 samples; concentration range 5–1477 ng/mL), sweat (1 case of overdose; 53 ng/patch), saliva (1 case of overdose; 5–18 ng/mL), hair (32 samples, including foetal hair; concentration range 0.20–21.56 ng/mg), but not in blood. EDEE was further suggested as a possible additional forensic marker for the particular situation of ‘crack’ and ethanol co-consumption [34].

#### 2.5.4. Excretion

Following metabolism, cocaine and its main metabolites are excreted in urine. EME and BE constitute the major excretion products, irrespective of the route of administration (intravenous, inhalation and intranasal) [74]. Moreover, approximately 1–3% of cocaine metabolic products excreted in urine are those resulting from *N*-demethylation to a norecgonine base, such as NBE, in addition to EC [24]. A subject who drank a cup of the Peruvian (4.14 mg cocaine) or Bolivian teas (4.29 mg cocaine) had, in their urine, a BE concentration of 3940 ng/mL and 4979 ng/mL, 10 and 3.5 h after ingestion, respectively [58].

Huestis et al. carried out a study resorting to 6 human subjects, where they investigated the urinary excretion pattern of cocaine and of some metabolites (BE, EME, *m*-OH-BE, *p*-OH-BE, NBE and EC), following smoking [33]. This study demonstrated a dose-dependent increase of the Maximum Concentration (C_max_) of all analytes, while the parameter Time-to-Maximum (T_max_) failed to show direct proportionality. Among the metabolites, EME presented the longest detection time (up to 164 h after a 40 mg dose). Another work by the same group assessed the urinary excretion of ecgonine and five other metabolites (BE, EME, *m*-OH-BE, *p*-OH-BE, NBE) following controlled inhalation, oral, intravenous, and intranasal administrations of cocaine, demonstrating that the route of administration does not significantly impact on C_max_ or T_max_ [35]. On the contrary, Cone et al. performed a study in 6 healthy male volunteers who were administered nearly equipotent doses of cocaine intravenously, through smoking and intranasally, and demonstrated that the elimination half-lives for cocaine and metabolites (BE, EME, NCOC, NBE, *m*-OH-BE, *p*-OH-BE, *m*-OH-COC and para-hydroxycocaine) were typically quicker for inhalation, intermediate for intravenous administration, and were the longest after intranasal administration [31].

### 2.6. Pharmacodynamics

Cocaine has different pharmacodynamic properties that make possible its use as a local anaesthetic and as a sympathomimetic stimulant at the central nervous system (CNS).

**Figure 3:**
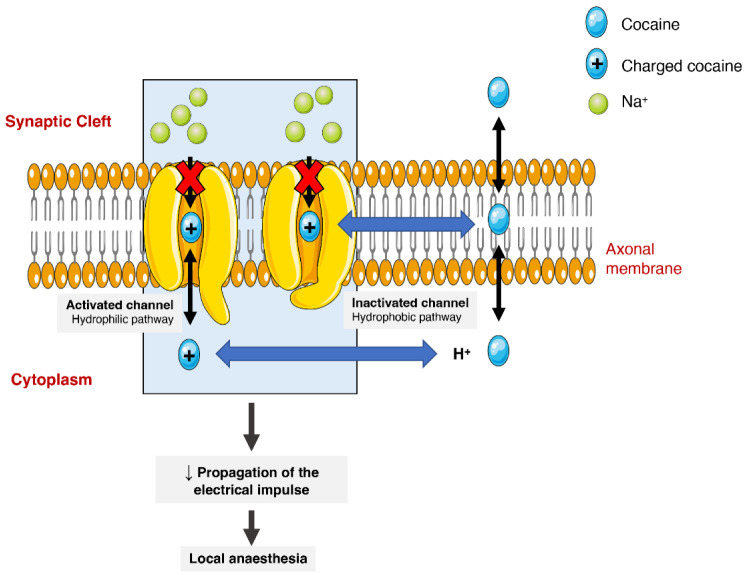
Schematic representation of cocaine’s interaction with voltage-gated sodium channels. Cocaine enters the channels and binds to them by two pathways (hydrophilic and hydrophobic). In the hydrophobic pathway cocaine interacts with the sodium channel at the membrane level, alternatively in hydrophilic pathway, the cocaine is ionized in cytoplasm before the interaction. In both cases, the flow of sodium is blocked, which diminishes the propagation of electrical impulses and causes a local anaesthetic effect.

The anaesthetic action of cocaine is related to its capacity to block voltage-gated sodium channels by stabilizing these channels in an inactive state (Figure 3). The binding of cocaine to the channel’s pore prevents sodium from flowing through it into the cells and thus blocking the depolarization process and the propagation of the electrical impulses [1,24,75]. The current medical use is very limited as most countries consider it obsolete. It can still be used as a topical anaesthetic, which might be particularly useful for endoscopic sinus surgery, given its vasoconstrictive effects. There are, however, controversies related to the development of mild morbidities, such as hypertension and tachycardia [76].

On the other hand, the psychoactive and sympathomimetic effects of cocaine derive from the blockade of presynaptic transporters responsible for the reuptake of serotonin, noradrenaline, and dopamine. In the case of the latter, the blockade of the presynaptic dopamine transporter (DAT) in the synaptic cleft causes an extracellular increase in dopamine with an overstimulation of the dopaminergic postsynaptic receptors, inducing the euphoric ‘rush’ [3,53]. Further mechanisms of tolerance at this level are responsible by the subsequent drop in the dopamine levels experienced as a dysphoric ‘crash’. A recent meta-analysis showed that chronic cocaine users display a significant reduction in dopamine receptors D_2_ and D_3_ in the striatum, the caudate and putamen brain regions, as well as a significantly increased availability of DAT all over the striatum [77].

**Figure 4:**
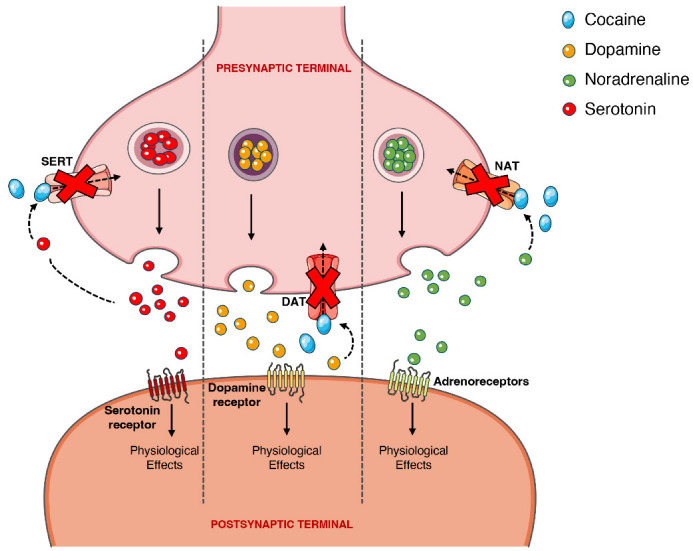
Schematic representation of cocaine’s pharmacodynamics at the noradrenergic, serotonergic or dopaminergic synapse. Cocaine acts by blocking the presynaptic transporters of dopamine, serotonin and noradrenaline, preventing the reuptake of the neurotransmitters into the presynaptic terminal, which will cause intense and prolonged stimulation of the postsynaptic receptors. **DAT,** dopamine transporter; **NAT,** noradrenaline transporter; **SERT**, serotonin transporter.

When cocaine is consumed, an exacerbated dopaminergic activity along the mesocorticolimbic pathways occurs. Neurons from these pathways are located in the ventral tegmental area and project to other brain locations, including the nucleus accumbens [78]. This could explain why the drug has such an addictive potential, since it is well acknowledged that the nucleus accumbens may have an important role in the rewarding and addictive properties of cocaine and other drugs [79]. However, it should be mentioned that cocaine’s capacity to increase serotoninergic activity (which may induce seizures) could also contribute to the drug’s addictive potential [80,81]. Figure 4 schematically represents cocaine’s pharmacodynamic action over the monoaminergic system.

The sympathomimetic properties of cocaine are related to the above-mentioned inhibition of noradrenaline reuptake via noradrenaline transporter (NAT). Because cocaine impedes this reuptake of noradrenaline, and thus increases its availability, there will be an increase in the stimulation of the α- and β-adrenergic receptors, and an augmented adrenergic response—which relates to the marked vasoconstrictive properties of the drug (responsible for a few of the cardiotoxic effects) [2,82,83].

Additionally, cocaine also has the capacity to directly target adrenergic, *N*-methyl-*D*-aspartate (NMDA), and sigma and kappa opioid receptors. Cocaine affects NMDA receptors, as exposure to the drug modulates (for greater or lesser) receptor subunit expression, alters receptor distribution in the synapse, and influences the crosstalk of the NMDA receptor with the dopaminergic receptor D_1_, in different brain areas, for example, the nucleus accumbens, the ventral tegmental area and the prefrontal cortex [84]. Lastly, cocaine acts directly over the sigma opioid receptors, binding with greater affinity to the σ_1_ receptor than to the σ_2_ receptor; agonism at the σ_1_ by cocaine partially mediates the hyperlocomotion and seizures, and these receptors are paramount in the establishment of cocaine-induced conditioned place preference in mice [53,82,85].

Recently, it has been suggested that the pharmacological action of cocaine over DAT may not be as simple as the sole inhibition of the transporter’s reuptake function, as its behaviour is distinct from other DAT inhibitors of equal or greater potency (with matched capacity for crossing the BBB) and resembles methylphenidate (a norepinephrine-dopamine reuptake inhibitor that also induces the release of synaptic dopamine). As such, it was hypothesised that, similar to amphetamines, cocaine functions as a negative allosteric modulator of DAT (i.e., a DAT ‘inverse agonist’), altering transporter function and reversing transport direction [86]. However, more research is necessary in this area to further clarify cocaine pharmacodynamics.

### 2.7. Effects and Toxicity of Cocaine

Cocaine’s LD_50_ has been previously determined in a few studies using different animal models: in mice, using an intraperitoneal administration, it was valued at 95.1 mg/Kg [1]; in rats and dogs using an intravenous route, the values were 17.5 and 21 mg/Kg, respectively [53].

As previously stated, cocaine targets the CNS, inducing a myriad of physical, psychological, and behavioural effects, which are inherently dependent on the user’s profile, route of administration and dose. While many of the severe pathological effects induced by cocaine could be attributed to a chronic consumption pattern (e.g., neurodegeneration, premature brain aging, depression, blood vessels damage), certain effects, such as tachycardia, hypertension, hyperthermia, diaphoresis, tremors, seizures, mydriasis, headaches, abdominal pain, muscle hyperactivity, haemorrhagic stroke, and multiorgan failure, arise with acute abuse patterns (all too often, even after a single dose). It is important to keep in mind that some cocaine metabolites maintain the ability to cross the BBB, thus contributing to both desirable effects and adverse/toxic reactions reported by users [54].

#### 2.7.1. Subjective and Physiological Effects

Moderate doses of cocaine induce euphoria, improve alertness and concentration, increase libido, promote a general sensation of well-being, and reduce fatigue and appetite. This is, however, accompanied by insomnia, anxiety, irritability, dysphoria, and impulsive behaviour—the less desirable effects may not be noticed immediately and might increase in frequency with continued drug use. Hallmark physiological effects of cocaine include those of the cardiovascular nature, such as vasoconstriction, tachycardia, and hypertension. Moreover, hyperthermia, diaphoresis, tremors and seizures, mydriasis, headaches, abdominal pain, and muscle hyperactivity may also arise, compromising furthermore the health of the user, and potentially leading to convulsions and/or cardiovascular and respiratory failure [83,87,88].

#### 2.7.2. Hyperthermia

The use of cocaine is associated with hyperthermia, which represents one of the most clinically relevant aspects in the drugs’ toxicity as the high body temperature can cause disseminated intravascular coagulation, rhabdomyolysis, and other multi-organ toxic events (‘heat infarct’) [89]. In fact, cocaine-induced hyperthermia potentiates the risk of user’s death at plasmatic concentrations 10–20 times lower than the average fatal level (~6 mg/L) [90]. Hyperactivity induced by cocaine leads to a further increase in body temperature; in addition to this, the vasoconstrictive effect of the drug also contributes to a generalised rise in the user’s body temperature, by limiting dermal blood flow and impairing heat dissipation [89,91,92]. Activation of dopaminergic and serotoninergic receptors is postulated to contribute towards the hyperthermic effects of cocaine: in a recently published work, the dopaminergic-serotoninergic antipsychotic risperidone, the serotonin 2A receptor antagonists ritanserine and ketanserine, as well as selective dopaminergic antagonist haloperidol and selective D_1_-antagonist SCH23390 were capable of reverting cocaine-induced hyperthermia in Wistar rats intraperitoneally administered with a 30 mg/Kg dose [93]. The same work additionally demonstrated that the cocaine bolus increased the levels of dopamine, noradrenaline, and serotonin in the hypothalamic thermoregulatory centre of the body, resulting in an impairment of the body temperature set point and an imbalance of heat production and dissipation mechanisms. Furthermore, the frequent co-consumption of alcohol alongside cocaine contributes to dehydration and to a decrease in sweat production, adding to the difficulty in keeping the body’s temperature regulated [92], which is further aggravated by the consumptions settings (e.g., the drug is often consumed in crowded and hot places associated with the continuous dancing without sufficient rest or rehydration) [94].

#### 2.7.3. Cardiovascular System

The cardiovascular system is particularly susceptible when it comes to cocaine toxicity. Typical cardiovascular manifestations related to cocaine use are hypertension, tachycardia, and ischemia, but more severe consequences are also frequent. They include acute myocardial infarction, dysrhythmias, aneurysm, accelerated atherosclerosis, cardiomyopathy, decreased left ventricular function and heart failure [95,96].

**Figure 5:**
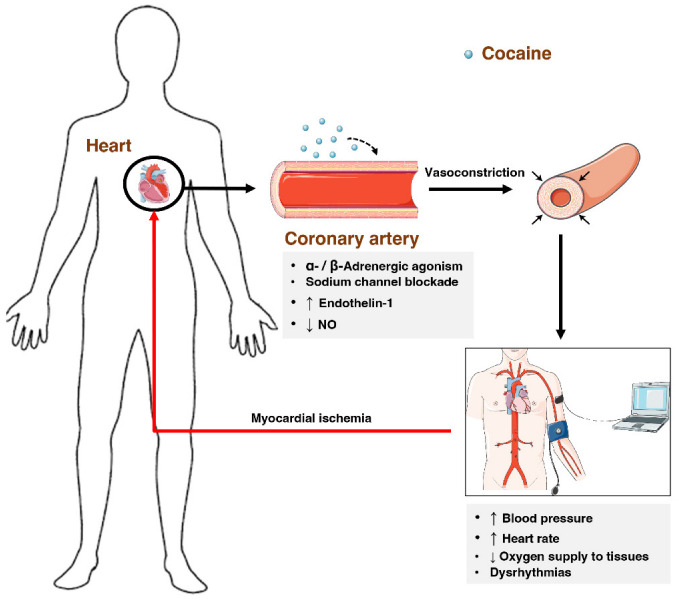
Influence of cocaine over the cardiovascular system. Cocaine promotes vasoconstriction, through indirect agonism of α-/β-adrenergic receptors, blockade of voltage-gated sodium channels, and increases in endothelin-1 and decrease of nitric oxide. These factors will increase the heart rate and blood pressure, decrease the supply of oxygen to tissues, and ultimately induce dysrhythmias.

The literature reports on numerous mechanisms to explain the toxicity of cocaine at the cardiovascular level. Firstly, by interfering with the reuptake of catecholamines and indirectly acting over α- and β-adrenergic receptors, cocaine can induce vasoconstriction of the coronary arteries and markedly increases oxygen demands by speeding up the heart rate and stimulating contractility of the heart. Moreover, the induced increase of endothelin-1 (a vasoconstrictor) and reduction in the production of nitric oxide (a vasodilator) creates an imbalance that favours vasoconstriction [96]. Consequently, oxygen supply to tissues decreases, with myocardial ischemia and acute myocardial infarction as possible outcome (Figure 5) [97,98].

As previously mentioned, the drug has the capacity to block the nerve’s voltage-gated sodium channels, thus preventing the conduction of the nervous impulse. This blockage compromises intracardial signal conduction, which results in a prolonged QRS interval, leading to dysrhythmia [95,96]. Additionally, a delay in ventricular depolarization ensues, ultimately causing a decrease of the left ventricular function [99]. Furthermore, the inhibition of the sodium currents could lead to a distortion of sodium-calcium extra-intracellular exchange with a subsequent decrease in the contractility of cardiomyocytes, due to low calcium cardiomyocyte cytosolic concentrations (Figure 3) [75].

Generation of reactive oxygen species (ROS) and oxidation products such as aminochromes and free radicals due to catecholamine oxidative metabolism, may also contribute towards cardiotoxicity, as increases in oxidative stress have been linked to apoptosis and cellular malfunction in the heart [83,100].

#### 2.7.4. Respiratory System

The respiratory system is also vulnerable to cocaine’s effects. The existing literature details that inhalation of ‘crack’ induces acute alterations in the respiratory tract, which does not occur when the same dose of the drug is administered intravenously [101]. Respiratory complications associated to cocaine use are, for example, bronchoconstriction, pneumothorax, pneumomediastinum, pulmonary hemorrhage, non-cardiogenic pulmonary edema, asthma exacerbation, among others [82,102,103]. These effects are not only due to a local irritant effect that induces bronchospasm, but also arise from the lungs exposure to cocaine vapor with toxic products deriving from cocaine pyrolysis (e.g., AEME), impurities, adulterants of ‘crack’ (such as caffeine, lidocaine and prilocaine) and combustion products [54,83].

The vasoconstrictive properties of cocaine also affect the respiratory system, particularly at the nasal level for intranasal administration. The cocaine-induced midline destructive lesion occurs because of the continuous vasoconstriction, which the vessels in the nasal lining mucosa are subjected to when users ‘snort’ cocaine [3]. Prolonged vasoconstriction of the tissue leads to the development of ischemia and, in conjunction with the inflammatory process, ultimately results in the perforation of the nasal septa [3].

‘Crack lung’ is an acute pulmonary syndrome characteristic of individuals who select smoking as their preferred route of cocaine administration. A typical ‘crack lung’ case will manifest as chest pain, fever, hemoptysis, and hypoxemia associated with acute pulmonary infiltrates [54,83]. Additionally, the lung may have an anthracotic appearance due to the accumulation of carbon in macrophages and coughed-up secretions may be black [3].

All mechanisms involved in pulmonary alterations are not fully known. However, the pulmonary vasculature presents adrenergic receptors that can be activated by excessive catecholamine activity. While the stimulation of α1-adrenergic receptors is related to contraction of bronchial capillaries, the activation of β2-adrenergic receptors induces bronchial muscle dilation. Of note, non-cardiogenic pulmonary oedema may occur due to damage of the endothelium of pulmonary vessels, which will also increase their permeability [83].

#### 2.7.5. Renal System

Cocaine can induce acute kidney failure, in particular with a chronic abuse pattern. There are many factors that can instigate cocaine-related kidney injury, involving oxidative stress, renal atherogenesis, changes in glomerular matrix synthesis and local hemodynamic changes [83]. In addition, rhabdomyolysis, renal infarction, vasculitis, acute interstitial nephritis, thrombotic microangiopathy and malignant hypertension are also often associated causes [104,105]. Each of these events has underlying causes. The development of rhabdomyolysis may occur: (1) in response to hyperthermia with subsequent release of myoglobin that may cause cytotoxicity in the kidney cells causing acute tubular necrosis; (2) as a consequence of the vasoconstrictive effects of the drug—causing muscle ischemia and necrosis; (3) by direct toxicity resulting in skeletal myofibrillar degeneration; the formation of free radicals can also contribute towards this [105]. Renal infarction is related to increased thromboxane production, platelet aggregation, vasoconstriction, and matrix accumulation [83,104].

A few studies have explored the mechanisms of nephrotoxicity of cocaine at the cellular level. In primary cultured human proximal tubular epithelial cells, cocaine at 5 mM (arguably an extremely high dose not likely to be found in users’ bodies) caused a decrease in cellular viability after 48-h exposure and impacted intracellular adenosine triphosphate (ATP), while 0.5 mM were enough to diminish reduced glutathione (GSH) levels [106]. Furthermore, this same study demonstrated that cocaine concentrations between 0.1 and 2.5 mM induced an increase in apoptotic cells, and necrotic cells appeared following 5 mM cocaine exposure. An in vivo study, where mice were administered with 60 mg/Kg cocaine via IP per day, reported increases in oxidative stress demonstrated through several findings such as enhanced lipid peroxidation and protein oxidation, decrease in the ratio of reduced/oxidized glutathione, reduced activity of glutathione reductase and peroxidase and increased superoxide dismutase (SOD) activity, as well as changes in the expression of anti- and pro-apoptotic proteins. Histopathological changes such as focal tubular necrosis, hemorrhage and congestion, tubular epithelial vacuolization, and interstitial mononuclear cell infiltration and greater tubulointerstitial injury were observed [107]. A recent study exploring cysteine metabolism in cocaine self-administering rats found that 185 mg/Kg of cocaine (intravenous) led to an increase in reactive sulfur species in kidneys, which remained significantly high following 10-day abstinence, indicating that cocaine shifted cysteine metabolism to an anaerobic pathway [108]. The same research group published a previous work in which rats administered 10 mg/Kg of cocaine also shifted cysteine metabolism—a single dose of cocaine led to increased sulfane sulfur whole pool, decreased bound sulfane sulfur and levels of ROS and glutathione-S-transferase, while a repeated dose regime (5 days) induced a decrease in hydrogen sulfide and caused an increase in sulfane sulfur whole pool and lipid peroxidation [109].

#### 2.7.6. Brain

Cocaine and crack use also have a myriad of repercussions for the brain. The use of these drugs is associated with the occurrence of ischemic and hemorrhagic stroke, the increase in arterial blood pressure being the main culprit, although interferences with normal hematological parameters induced by cocaine may also play a role [54,89]. The occurrence of intracranial and subarachnoid hemorrhages is equally related to the dysregulated increase in blood pressure [83,110]. Due to the increase in blood pressure, as well as the effect of cocaine over serotoninergic and dopaminergic systems, headaches are also common [110]. Seizures also occur frequently. They arise not only in chronic users but also after a single dose, as cocaine has the capacity to lower seizure threshold, through a chronic low intensity stimulation of the limbic system (kindling) [83,89]. The blockade of noradrenaline by cocaine is also a contributing factor for this increased seizure occurrence. Of note, a recent work determined that cocaine’s kindling effect, which is related to a significant increase in p53 expression in the brain, can be attenuated by p53 genetic depletion [111].

In vivo and in vitro studies have also shown that cocaine has a neurotoxic potential. Cunha-Oliveira et al. saw that 1 mM of cocaine led to an increase in calcium concentrations and caspase-3 activity, as well as a decrease in mitochondrial membrane potential and ATP in rat cortical neurons exposed for 24 h [112]. Furthermore, cocaine exposure in models of rat primary hippocampal neurons (1 mM) and mouse primary cortical neurons (1, 10, 100 and 200 μM) increased the expression of autophagy markers LC-3 I and II [113,114]. Nifedipine, a selective blocker of L-type calcium channels, reverted the reduction of cerebral blood flow and tissue oxygenation induced by increases in neuronal calcium currents, in the prefrontal cortex of rats exposed to 1 mg/Kg cocaine [115]. Increased oxidative stress is another mechanism contributing to cocaine’s neurotoxic effects [83]; for example, one study investigating cocaine’s effects in rat cerebellum proved that, after 18 days of a 15 mg/Kg administration, the drug increases oxidative stress, by decreasing the activity of glutathione peroxidase (GPx) as well as reducing the reduced to oxidized glutathione ratio, and by increasing the concentration of glutamate, nuclear factor kappa B and CD68, indicating microglial-macrophage activation [116].

Morphological differences between users and non-users of cocaine have also been investigated. One research delving into the grey matter abnormalities of crack users discovered diminished cortical thickness in the left temporal, orbitofrontal and rostro frontal cortexes and reduced grey matter volume in the right hippocampus and ventral diencephalon [117]. The left and right nucleus accumbens, a brain area crucial in reward, pleasure, and reinforcement learning processes, has also been proven to have a reduced volume in crack users compared to healthy controls (with no differences in intracranial volume) [118].

#### 2.7.7. Liver

Cocaine is a well-known hepatotoxic substance. This was first demonstrated in humans in 1987, when the presence of inflammation and periportal necrosis with moderate infiltration of lipids was verified—prior to this, hepatotoxicity was reported solely in animal models. This first case was consistent with earlier and later studies [119,120,121]. The hallmark hepatic lesion following cocaine use is hepatocellular necrosis, which was also demonstrated in animal studies [83,122]. Other pathological characteristics of cocaine-induced hepatic injury include increased infiltration with fatty acids, increased blood aspartate aminotransferase levels and pernicious conjugates of reactive cocaine metabolites with cellular macromolecules [83].

Research has shown a connection between cytochrome P450-mediated cocaine bioactivation and the inhibition of hepatic metabolism, with the drug’s hepatotoxic properties. After the formation of NCOC (also catalyzed by flavin adenine dinucleotide-containing monooxygenases), it is oxidized in the liver, leading to the generation of oxidative metabolites (such as *N*-hydroxynorcocaine (N-OH-NCOC) and norcocaine nitroxide (NCOC-NO^•^)). The subsequent oxidation of NCOC-NO^•^ forms a highly reactive cation, norcocaine nitrosonium, which binds in an irreversible manner to cellular proteins and causes cell death. Additionally, NCOC-NO^•^ can also be reduced toN-OH-NCOC, contributing to the formation of free radicals, which will induce oxidative stress and ultimately result in cell death [62,122,123].

Cocaine’s ability to interfere with the hepatocytes’ antioxidant system (e.g., SOD, GPx, catalase) and to depress mitochondrial respiration is well-known. A recent work by Mai et al., investigating the protective potential of GPx, exemplifies the cellular damage of cocaine (at 60 and 90 mg/Kg BW) in an in vivo study using mice. There were significant increases in SOD activity, ROS, protein carbonylation and lipid peroxidation, cleaved caspase-3 expression and intramitochondrial calcium, accompanied by a significant decrease in GPx. Examples of histological changes include hemorrhage, congestion, periventricular necrosis and hydropic degeneration [124]. Notably, enhanced expression of GPx protected the animals from having such severe outcomes, as did the depletion of p53 [125]. Another study investigating the influence of cocaine and its N-oxidative metabolites over mitochondrial respiration, determined that while cocaine had no influence over respiration states 3 and 4 or respiratory control ratio, the metabolites NCOC, *N*-hydroxycocaine and NCOC-NO^•^ did impact mitochondrial respiration. It was therefore suggested that these metabolites are in fact responsible for the depletion of intracellular stores of ATP and ensuing cell death [126]. Furthermore, cocaine can stimulate a cascade of reactions, including caspase-3 activation and cytochrome c release, leading to hepatocellular apoptosis [62]. The study by Kowalczyk-Pachel et al., where rats self-administered cocaine (to the maximum of 185 mg/Kg), found that, compared to control animals, cocaine decreased the levels of reactive sulfur species, and sulfate levels were also impacted; similar to effects in kidneys, these alterations remained even during the abstinence period, and reflected the impact of cocaine over cysteine metabolism [108].

One recent study used a metabolomics approach to study the effects of cocaine in the HepG2 hepatoma cell line, after exposure to 200 mM of the drug. Significant alterations to amino acid metabolic pathways (e.g., glycine, serine, threonine, arginine, proline, taurine) were observed, which were most marked in the cases of glutamate, aspartate and alanine [127]. A previous study, using a more metabolically competent model (Sprague-Dawley rats), analyzed plasma of cocaine-addicted animals (which were given a dose of 10 mg/Kg) and found significant changes to the levels of L-threonine, spermidine, cysteine and n-Propylamine [128].

### 2.8. Abuse Potential, Dependence, and Tolerance

The abuse and dependence of cocaine is strongly related to the drug’s capacity to induce the release of dopamine within the mesocorticolimbic circuit (also known as the reward system). As the user continues to consume cocaine, desensitization occurs and so larger doses are necessary to induce stimuli of the same magnitude as before, as well as to minimize withdrawal symptoms [129]. Cocaine dependence/addiction specifically is not included in the Diagnostics and Statistics Manual of Mental Disorders 5th edition (DSM-5); however, the criteria for stimulant use disorder can be applied. The criteria set for this are: hazardous use, neglected major life roles (e.g., work, parenting) to use, social/interpersonal problems related to use, craving, withdrawal, tolerance, activities given up to use, much time spent using, used larger amounts/longer, physical/psychological problems related to use, and repeated attempts to quit/control use [130].

Cocaine has been demonstrated to possess an elevated abuse potential, with experimental studies reporting it induces place preference conditioning and readily acts as reinforcer for drug self-administration [131,132,133,134]. Di Chiara and Imperato tested the effect of cocaine on extracellular dopamine content in two terminal dopaminergic brain areas of rats (the dorsal caudate nucleus and the nucleus accumbens septi), and found that the drug has the capacity to increase dopamine concentrations in both the areas, but especially in the nucleus accumbens, postulating that this ability could be a key element of drugs of abuse [135]. The dorsal striatum also seems to be involved in cocaine dependence, given that in dependent individuals, the exposure to cocaine cues (a video of subjects consuming ‘crack’) reduced the binding of a radioligand to D2 receptor in this brain region, and greater displacement of the radioligand corresponded with craving. Subjects with the highest degrees of withdrawal and addiction also had the greatest degree of displacement [136]. Furthermore, Volkow et al. determined that, when compared to non-dependent individuals, cocaine-dependent subjects demonstrate impaired dopamine increases in the dorsal and ventral striatum in response to methylphenidate, which did not differ from that elicited by the placebo. This same study found that the baseline levels of dopaminergic D2 and D3 receptors of the ventral striatum were markedly lower for cocaine abusers, [137]. Recent advances in the field revealed that the heteromerization of receptors D2-NMDA induced by a cocaine regimen in mice was sustained after an abstinence period, and was associated with behavioral sensitization by the drug [138]. Furthermore, D2-NDMA heteromeric complexes were demonstrated to be necessary for the development and reinstatement of conditioned place preference induced by cocaine, and inhibiting their formation did not interfere with natural reward processes [138].

‘Crack’ dependence has been proven to affect working memory: ‘crack’-dependent young women performed similarly to healthy older women, in an inferior manner to younger healthy women (for both groups) [139]. It seems clear that, while a fuller and more complete picture of the mechanisms that underlie cocaine abuse and dependence is beginning to form, more research is still necessary to better help those struggling with cocaine addiction.

The continued use of cocaine at high doses can lead to the development of tolerance to the cardiovascular and subjective effects reported by users, with cocaine-dependent volunteers who underwent continuous infusions describing a subdued ‘rush’ as time passed, but still feeling the ‘high’ [140]. In fact, one study approaching long-term cocaine users in Philadelphia and applying the ‘Cocaine History Questionnaire’ found that there was a negative correlation between the amount of cocaine consumed and the sensation of euphoria achieved from the use, while some negative effects (mood swings, paranoia and agitation) associated with the use increased [141]. Animal studies have also helped to shed some light regarding cocaine tolerance. At the pharmacodynamic level, cocaine self-administration at 1.5 mg/Kg (40 injections per day for five consecutive days) reduced the amount of dopamine and the velocity at which the neurotransmitter is released, as observed in rat brain slices [142]; this same treatment led to a reduction in effect of several dopamine-noradrenaline uptake blockers (bupropion and nomifensine), but did not affect response to dopaminergic releasers (e.g., methamphetamine and phentermine). Furthermore, the same regimen of cocaine intake led Sprague Dawley rats to increase the number of self-administrations within the first hour of the session over five consecutive sessions, and a tolerance for the locomotor-activating effects of cocaine [143]. In addition, the self-administration of cocaine caused a reduction in the amount of presynaptic dopamine and its uptake in the nucleus accumbens, and DAT showed a reduced sensitivity to cocaine’s capacity to inhibit dopamine uptake [143]. The development of tolerance—where the pleasurable effects of the drug are diminished—could lead the individual to feel the need to administer a new bolus (increase the dose and/or intake frequency) while plasma concentrations are still elevated, and thus increasing the likelihood of severe and even possibly fatal toxicity [2,96,144].

### 2.9. Polydrug Use

Adulterants and contaminants are often present in cocaine samples, as indicated by analysis of a pool of samples acquired in the street that averaged 40%. Many of these additives are often included to increase the perceived volume (e.g., talc, sugar or corn starch) or purity of cocaine (e.g., lidocaine, benzocaine, and procaine; caffeine, ephedrine) and may modulate cocaine’s biological effects, including toxicity [24]. In addition to these substances, polydrug use with both licit and illicit drugs is a common practice among cocaine users [2,145,146,147,148]. Polydrug use constitutes a risk for users for a myriad of reasons, including the potentiation of noxious effects of one drug by the other(s) due to the formation of new (and perhaps more toxic) metabolites and/or the competitive inhibition of metabolizing systems. The choice of the drug to combine with cocaine is often based on the desire to counteract the stimulant (‘upper’) effects of cocaine, so another drug to ‘mellow down’ (a ‘downer’) is frequently selected. Examples of these drugs are alcohol, benzodiazepines (e.g., lorazepam and diazepam), cannabis and opioids (e.g., heroin) [149]. Two of the most common combinations are cocaine in conjunction with alcohol and opioids/heroin (also known as ‘speedball’) [1,24], and therefore will be given special standout.

#### 2.9.1. Alcohol

A vast majority of cocaine users co-consume it with alcohol, and report that this combination extends the duration of the stimulation and counterbalances the dysphoria subsequent to cocaine use [24]. Generally, ethanol potentiates both the morbidity and mortality of cocaine [150,151]. The use of cocaine in combination with alcohol is cardiotoxic [100] and leads to the formation of CE, a pharmacologically active metabolite, as previously mentioned. CE appears to be more selective for DAT than cocaine itself; CE is also capable of inducing an increase in blood pressure and heart rate, and it seems to enhance the effects cocaine has at the level of the CNS [152]; CE also possesses a longer half-life compared to cocaine and is capable of inhibiting the conversion of cocaine into BE. All these factors contribute to a more durable and thus simultaneously more dangerous stimulation [24,123,152]. One recent study attempted to establish a relationship between blood concentrations of cocaine and CE and the severity of clinical manifestations among individuals hospitalised due to cocaine intoxication [153]. The mean blood concentrations of cocaine and CE were 0.34 ± 0.45 μg/mL and 0.38 ± 0.34 μg/mL, respectively, and while it was not possible to establish a pattern between patient prognosis or their treatment course with the blood concentrations of these substances, the evaluation could be helpful to indicate the severity of the intoxication.

#### 2.9.2. Heroin/Opioids

The co-use of cocaine and heroin is commonly known as ‘speedball’. In this combination, the heroin and cocaine can be administered in a mixture, or the cocaine may be administered immediately before or after heroin [146]. Both drugs compete for the same enzymes, such as hCE, in the metabolic process, which can prolong their biological effects. The social and health-related consequences of the use of cocaine by opioid-dependents seem to be particularly negative, due to the high frequency of use through the intravenous route and the short half-life of cocaine, which drives up the number of times the user injects. Furthermore, the practice of sharing syringes contributes to the spread of infectious diseases [146]. The EMCDDA 2021 report on drug-associated deaths in Europe discloses that opioids are the most commonly found drug in cocaine-related deaths [52]. Despite its capacity to moderately increase cerebral oxygen levels, cocaine does not impact brain hypoxia induced by high doses of heroin [154]. The motives for this co-use differ in accordance with the user’s goals: to experience the unique effects of the combination of heroin and cocaine (this stimulation is different from either drug alone), to attain greater euphoric effects, or even to be able to reduce heroin use and the withdrawal symptoms [146].

### 2.10. Management of Acute Intoxications and Cocaine Use Disorder

#### 2.10.1. Treatment of Acute Intoxication

Determining the ‘lethal dose’ of cocaine is difficult, due to the high degree of variability associated with the manner in which users react to cocaine intake and metabolize it, due to the variations in the metabolic rate of individuals, potential drug interactions and genetic polymorphisms of metabolizing enzymes [96]. Furthermore, there seems to be no relation between blood concentrations and toxicity, with plasma cocaine concentrations of 0.029 mg/L found in a patient who died, and few symptoms occurring in an individual with 3.9 mg/L (a concentration previously considered nearly fatal) [155]. Comparatively, in Swiss-Webster mice, cocaine’s LD50 was estimated at 93 mg/Kg [156].

A severe cocaine intoxication can result in a fatal outcome if not given the necessary medical treatment [157]. Given the ever-present risk of cardiorespiratory arrest, monitoring vital signs is extremely important, and cardiorespiratory resuscitation should be performed as soon as necessary. If this fails, the administration of vasopressin is recommended (this therapeutic option has demonstrated greater effectiveness than epinephrine, the first-line drug for cardiac resuscitation) [158,159].

Benzodiazepines are useful in the treatment of subjects who, in addition to showing signs of myocardial ischemia, are anxious, tachycardic or hypertensive. Not only do benzodiazepines exert anxiolytic action, but they also attenuate toxic effects at the cardiovascular and cerebral level, by reducing both blood pressure and cardiac output, which makes them a key first approach in treating cocaine acute intoxications [96,160]. Of note, when the subject rejects benzodiazepines’ oral administration, the intramuscular or intravenous routes are recommended [89,160].

In some cases, even after receiving the administration of oxygen, aspirin, benzodiazepines, and nitroglycerin, the subjects still have chest pain. In these instances, administration of the non-selective α-adrenoceptor antagonist phentolamine is recommended to induce vasodilation, as β-adrenergic blockers are not useful in treating this clinical manifestation [2,96]. While the usefulness of calcium channel antagonists in the treatment of cocaine-related chest pain is not fully known, verapamil is considered effective in reversing the vasoconstriction, and should therefore be administered after the benzodiazepines to ensure some protection for the CNS [2,161].

The use of antipsychotics to manage cocaine intoxications is questionable and potentially dangerous, as they may intensify the risk of cardiac dysrhythmias. Furthermore, in the case of subjects medicated with other drugs, such as tricyclic antidepressants, there is a high risk of potentiation of these drugs’ effects [162]. For these reasons, the administration of antipsychotics should be considered with caution [163].

#### 2.10.2. Treatment of Cocaine Addiction/Dependence

Currently, no pharmacological therapies are approved in Europe or the United States for the management of cocaine use disorder (CUD). The most promising pharmacological strategies for treatment of CUD include the use of dopaminergic agonists, such as long-acting amphetamine or drugs capable of influencing glutamatergic and GABAergic systems such as topiramate [164]. The effectiveness of these treatments was evaluated in preliminary clinical trials. In the Netherlands, 73 patients with treatment-refractory heroin and cocaine dependence reported fewer days of cocaine use (45 days) after 12 weeks of oral administration of sustained-release dexamphetamine (60 mg/day) compared with placebo-treated patients (61 days) [165]. Regarding the use of glutamatergic/GABAergic medications, 170 cocaine- and alcohol-dependent individuals treated with topiramate (300 mg/day for 13 weeks) were significantly more likely to achieve abstinence from cocaine during the last 3 weeks of treatment [166]. Modafinil has also shown promising results in treating moderate CUD, as it can weaken cocaine-induced euphoria in humans; however, it is not effective in reducing cocaine intake if the subjects have an alcohol dependence in conjunction with CUD [167].

Psychosocial approaches remain limited but linger nonetheless as the treatment of choice for CUD, with standard approaches including contingency management and cognitive behavioural therapy. Although no figures on the rates of success of such approaches are available, a recent meta-analysis comparing treatment options for CUD in adults concluded that contingency management therapies were the only treatment positively associated with a reduction in the use of cocaine [168].

## 3. Conclusions

Cocaine remains to this day a matter of concern for public health, as it holds strong as the second most used illicit substance in most countries. Whether it is in the form of cocaine powder or ‘crack’ cocaine, its prevalence and use by individuals from all walks of life should be taken seriously as it will not spare users from the inherent toxicity of the drug’s use. This review will hopefully assist the reader in obtaining a global, clear, and more complete picture of what is known about cocaine toxicity, be this in terms of the analytical methods to detect it, the (non-)biological matrices where it can be detected, its pharmacokinetics and pharmacodynamics, the possible pathophysiological repercussions for users, and the existing courses of treatment for cocaine intoxication and CUD.

## Figures and Tables

**Table 1:** Physical and chemical properties of cocaine.

Cocaine Form	Cocaine Hydrochloride	Cocaine Free Base
**Other names it is known by**	‘coke’, ‘snow’, ‘blow’	‘crack’
**CAS**	53-21-4	50-36-2
**Molecular formula**	C_17_H_22_ClNO_4_	C_17_H_21_NO_4_
**Molecular weight**	339.8 g mol^−1^	303.35 g mol^−1^
**Boiling point**	-	187 °C
**Melting point**	195 °C	98 °C
**Solubility in water**	2 g/mL	1.7 × 10^−3^ g/mL
**pKa; pKb (at 15 °C)**	-	8.61; 5.59
**Log P**	-	2.3

## Data Availability

Not applicable.

## Abbreviations

AEME	anhydroecgonine methyl ester
ATP	adenosine triphosphate
BBB	blood brain barrier
BE	benzoylecgonine
CE	cocaethylene
CNS	central nervous system
CUD	cocaine use disorder
CYP450	cytochrome P450
DAT	dopamine transporter
DSM-5	Diagnostics and Statistics Manual of Mental Disorders 5th edition
EC	ecgonine
ED	ecgonidine
EDEE	ecgonidine ethyl ester
EEE	ecgonine ethyl ester
EMCDDA	European Monitoring Centre for Drug and Drug Addiction
EME	ecgonine methyl ester
FADM	flavin adenine dinucleotide-containing monooxygenase
GC-MS	gas chromatography coupled with mass spectrometry
GPx	glutathione peroxidase
GSH	reduced glutathione
hCE_1_	human carboxylesterase type 1
hCE_2_	human carboxylesterase type 2
HPLC	high performance liquid chromatography
LC-MS/MS	liquid chromatography coupled with tandem mass spectrometry
NAT	noradrenaline transporter
NBE	norbenzoylecgonine
NCE	norcocaethylene
NCOC	norcocaine
NCOC-NO^•^	orcocaine nitroxide
NCOC-NO^+^	norcocaine nitrosonium
NEDME	norecgonidine methyl ester
NEME	norecgonine methyl ester
NMDA	*N*-methyl-*D*-aspartate
N-OH-NCOC	N-hydroxy-norcocaine
(p-/m-)OH-BE	(para-/meta-)hydroxybenzoylecgonine
(p-/m-)OH-COC	(para-/meta-)hydroxycocaine
PChE	pseudocholinesterase
ROS	reactive oxygen species
SERT	serotonin transporter
SOD	superoxide dismutase
UPLC-MS/MS	ultra-performance liquid chromatography coupled with tandem mass spectrometry
